# Supplementary material for: The Use of Mechanical Ventilation Support at the End of Life in Motor Neurone Disease/Amyotrophic Lateral Sclerosis: A Scoping Review
Source: Brain Sci. 2022 Aug 30;12(9):1162. doi: 10.3390/brainsci12091162 (PMC9497211; doi:10.3390/brainsci12091162)
Supplement: Supplementary file 1 [file brainsci-12-01162-s001.zip › brainsci-1819994-supplementary.pdf]

Supplementary Materials

Table S1. Study characteristics

Author and date	Country	Type of evidence/study method	Relevant findings	Limitations for the purpose of review
Association for palliative Medicine of Great Britain and Ireland [25]	UK	Clinical Guidance	Provides background on the need for the guidance and current evidence. Provides guidance for clinicians in the form of five standards for the withdrawal of ventilation. Case examples used to illustrate key issues as well as a bibliography of available literature and direction for further reading and reference.	Withdrawal only
Baxter et al. (2013) The use of non-invasive ventilation at end of life in patients with motor neurone disease: A qualitative exploration of family carer and health professional experiences [13]	UK	Qualitative interviews with n = 9 bereaved family members and n = 15 nominated healthcare professionals referring to n = 10 patients. Thematic analysis was used	Relevant detail on keeping NIV in place until death, turning off the machine after death, need to document and share end of life wishes	Small sample of patient cases discussed (10 in total but 5 ceased NIV use prior to final stage)

Author and date	Country	Type of evidence/study method	Relevant findings	Limitations for the purpose of review
Berger. (2012) Pre-emptive use of palliative sedation and amyotrophic lateral sclerosis [53]	USA	Case illustration for discussion	Provides some insight into the planning of withdrawal and the withdrawal process itself. Use of pre-emptive sedation rather than in response to refractory symptoms at end of life. Conclude that this is an appropriate approach for the withdrawal of TV in this patient with MND.	Single case study presented, primarily debate, based on American guidance
Burkhardt et al. (2017) Is survival improved by the use of NIV and PEG in amyotrophic lateral sclerosis (ALS)? A post-mortem study of 80 ALS patients [42]	Switzerla nd	Retrospective autopsy study *n* = 80 (states 38/71 received NIV)	Some information on cause of death.72/74 died of types of respiratory failure. (6 assisted suicide). 38/71 received NIV. NIV increased risk of bronchopneumonia. Indicating NIV could be a risk factor, or prolonged survival may increase risk of pulmonary infection. Confirms increased survival time with NIV.	Limited information in relation to NIV. Not always clear if patients with NIV are included. Not clear if patients were using NIV when they died.
Cazzolli PA, Oppenheimer EA. (1996) Home mechanical ventilation for amyotrophic lateral sclerosis: nasal compared to tracheostomy-intermittent positive pressure ventilation [47]	USA	Prospective study of *n* = 75 patients (50 with TV and 25 with NIV). Interviews with patients and family members and some wider evaluative data gathered.	Small amount of information on deaths and reasons for withdrawal. Does include TV and NIV.	Age of the paper precludes the advances made in non-invasive ventilation. Methods not clearly described. Limited focus on end of life.

**Author and date**	**Country**	**Type of evidence/study method**	**Relevant findings**	**Limitations for the purpose of review**
Chapam et al. (2021) communication surrounding initiation and withdrawal of non-invasive ventilation in adults with Motor Neuron (e) Disease. Clinicians' and family members' perspectives [52]	Australia	Interviews with clinicians (*n* = 19), bereaved family members (*n* = 6) and a patient (*n* = 1)	Some focus on communication about initiating and withdrawal of NIV. Clinicians report challenges around when to have conversation, trying to assess 'family readiness' for such conversation and having the time to have these discussions properly. They also note patients' reluctance to discuss end of life and some challenges around having multiple teams involved. This can result in conflicting information and lack of responsibility for having difficult conversations. Family members recognised that they did not always want to discuss such issues but that they did expect this to be initiated by clinicians. Some felt if they did not ask end of life care would not be discussed.	
Dreyer et al. (2012) Withdrawal of invasive home mechanical ventilation in patients with advanced amyotrophic lateral sclerosis: Ten years of danish experience [11]	Denmark	Retrospective descriptive study of withdrawal of consent for TV over a 7 year period *n* = 12. One case example presented.	Focuses on the withdrawal of TV in 12 patients. Details how, when, where and the reasons for withdrawal taking place. Useful case study giving more detail on all aspects including planning to initiated TV and decisions about when it should be withdrawn. Once progressing to a total locked in state, in accordance with his advanced wishes he was sedated and ventilation withdrawn and death occurred shortly after.	Does not cover NIV or dying with ventilation in place.

Author and date	Country	Type of evidence/study method	Relevant findings	Limitations for the purpose of review
Eng. (2006) Management guidelines for motor neurone disease patients on non-invasive ventilation at home [61]	Australia	Literature review and stakeholder opinion in order to develop guidelines	One section on withdrawal presenting general information from the literature. Decisions should be made in advance, and with patient, family and clinicians involved. Infusion of morphine and midazolam should be used with additional intermittent doses for signs of distress (so not continuous deep sedation). Should be supervised by professional staff.	Focus on withdrawal only. Does not cover dying with ventilation on place.
Escarrabill et al. (2014)Place of death in patients with amyotrophic lateral sclerosis [40]	Spain	Retrospective medical records study of all deaths (*n* = 77) over a 12month period across 5 hospitals in Spain and Italy	Some information in relation to ventilation characteristics and place of death. More likely to die at home unless on facial ventilation, potentially due to increased care needs. 80% of those with TV died at home. This is also broken down by hours on ventilation. Greater number of hours on ventilation increases likelihood of dying in hospital. Access and use of medications was the same in both groups.	Does not correlate type of ventilation and amount of time using it with place of death. Not known in place of death was in line with patient's wishes
Faull and Oliver. (2016) Withdrawal of ventilation at the request of a patient with motor neurone disease: guidance for professionals [58]	UK	Review	Factual presentation of the guidance for UK professionals on withdrawal of ventilation and the reasoning behind this. Guidelines provide guidance on when to discuss withdrawal, the need to validate a patient's decision, that it should be undertaken within a reasonable time frame, the management of symptoms and the debrief of family members after the death.	Focus on withdrawal

Author and date	Country	Type of evidence/study method	Relevant findings	Limitations for the purpose of review
Faull et al. (2014) Issues for palliative medicine doctors surrounding the withdrawal of non-invasive ventilation at the request of a patient with motor neurone disease: a scoping study [41]	UK	Online questionnaire *n* = 130/993 doctors who were members of the Association of Palliative Medicine or Great Britain and Ireland	Focuses on the practical, emotional and ethical issues of withdrawal of NIV. 76/130 (58.5%) had been involved in withdrawal of NIV for MND patients. Those who had not been involved with withdrawal reported their expectation of the physical, ethical and emotional challenges to be higher than those who had taken part in with withdrawal. Practical challenges identified included preparations time, communication with the patient, how to manage symptoms and who should remove the mask. Ethical challenges were considered less that practical and emotional ones and included timing, appropriateness and interpreting ADRTs. Differences between causing and allowing death to occur were recognised but it was noted that is can 'feel' different for some. Emotional challenges included managing the emotions of others, supporting others, conflict resolution and death being related to an action.	Focuses on withdrawal of ventilation. NIV only.

**Author and date**	**Country**	**Type of evidence/study method**	**Relevant findings**	**Limitations for the purpose of review**
Faull and Wenzel. (2020) Mechanical ventilation withdrawal in motor neuron disease: an evaluation of practice [46]	UK	Prospective evaluation using a proforma of anonymised data from *n* = 37 clinical professionals to report on *n* = 46 patient cases who underwent withdrawal of ventilation.	Focus on medication use before, during and after withdrawal. Considerable variation in drug doses required. These were individualised according to patient need, primarily delivered via subcutaneous route. Time taken for the majority 2hrs or less, 10% much longer process up to 54hrs. Also report on response of family, with a small number of clinicians reporting families expressing grief beyond their normal experiences. Selection of useful quotes. Reflections on clinician's experience were generally positive.	Focus on withdrawal
Gannon. (2005) A request for hospice admission from hospital to withdraw ventilation [54]	UK	Case illustration and discussion	Discussion of the challenges face by hospice staff in accepting a patient, with whom they did not have a history, for the purpose of withdrawal. Effectively inheriting a decision made with a different healthcare team. Created conflict between continuity of care and professional obligation. Illustrates some of the emotive challenges for staff.	Focus on staff perspectives. Focus on withdrawal. Focus on NIV

**Author and date**	**Country**	**Type of evidence/study method**	**Relevant findings**	**Limitations for the purpose of review**
Gleeson and Johnson. (2017) Withdrawal of invasive ventilation in a patient with motor neurone disease and total locked-in syndrome [17]	UK	Case illustration and discussion	Ethical, legal and practical issues of withdrawing ventilation using a single case study. Illustrates the use of an advance care plan and MDT discussions in order to put the plan in place at the appropriate time. Describes the use of a 24hr continuous subcut infusion started 24hrs before withdrawal. Using 100mg Midazolam, 100mg diamorphine and 100mg levomapromazine. Patient had also specified who he wanted in the room, wife chose not to be there until his heart had stopped. Due to total locked in syndrome, heart rate was the only indicator of level of comfort. APM guidance on medication use in withdrawal not sufficient to cover those with TLS.	Focus on withdrawal of TV.
Kettemann et al. (2017) Clinical characteristics and course of dying in patients with amyotrophic lateral sclerosis withdrawing from long-term ventilation [43]	Germany	Observational cohort study $n = 49$	Assesses the use of augmented symptoms control verses continuous deep sedation for withdrawal of ventilation depending on level of dependence. ASC $n = 20$, CDS $n = 29$. As expected mean time until death was shorter in the CDS cohort. Both approaches considered useful and relevant. ASC may be considered closer to natural dying, but may be prolonging the process. CDS may be preferable to reduce stress for patients and families.	Focus on withdrawal and level of sedation. Both NIV and TV

**Author and date**	**Country**	**Type of evidence/study method**	**Relevant findings**	**Limitations for the purpose of review**
Kuhnlein et al. (2008) Palliative care and circumstances of dying in German ALS patients using non-invasive ventilation [38]	Germany	Structured interviews with n = 29 bereaved family caregivers	11/17 died peacefully with NIV in place. Only one report of 'choking' by a patient with NIV. 18 died at home and 11 in hospital. 69% had advance directives. GPs were involved with a third of patients, but less likely if in hospital. Sedatives, anxiolytics and opiates were used in a third of cases.	Not clear if some reported incidences are of patient's dependent on ventilation (9 reported poor tolerance or rare use of NIV). Higher focus on PAS and Euthanasia. Very small section on circumstances of dying. NIV only.

**Author and date**	**Country**	**Type of evidence/study method**	**Relevant findings**	**Limitations for the purpose of review**
Lebon and Fisher. (2011) Case report: Maintaining and withdrawing long-term invasive ventilation in a patient with MND/ALS in a home setting [55]	UK	Case illustration and discussion	Discussion of a single case of a man with TV. Clarifies the MDT role in decision making and facilitating withdrawal at the request of the patient. Subcut infusion was discussed and implemented to achieved sufficient levels of sedations (midazolam 30mg over 24hrs, increased to 60mg then 100mg), the patient remained alert and was frustrated with the delay so levomapromazine was added. PEG feeding was stopped. Once sedated the family gathered for final goodbye and only the patient's sister, community nurse, palliative care consultant and physio remained for the withdrawal. Died 15mins later. Legal and ethical distinctions need to be clear and some HCPs may wish to opt out of being involved.	Focus on withdrawal, focus on TV

Author and date	Country	Type of evidence/study method	Relevant findings	Limitations for the purpose of review
Markovic et al. (2018) Patterns of non-invasive ventilation in amyotrophic lateral sclerosis [44]	Canada	Retrospective cohort study<br>Patients from *n* = 48	Tracked NIV use over time and noted discussions on placement of a RIG, end of life discussions and DNAR order. NIV use increases over time. Considerable variability in timing of end of life discussion. Strong link between the discussion of resuscitation and establishing as DNAR and also between starting NIV and having a DNAR.	Content and context of end of life or DNAR conversations not reported. Not clear if patients were dependent on NIV when they died. Missing data from second ventilators, logging daytime use. No documentation of discussions about future ventilation use or withdrawal. Also note that end of life and DNAR discussions may not have been noted if no decision was made.

**Author and date**	**Country**	**Type of evidence/study method**	**Relevant findings**	**Limitations for the purpose of review**
Messer et al. (2020) Requested withdrawal of mechanical ventilation in six patients with motor neuron disease [18]	UK	Case illustration of *n* = 6 patients who requested withdrawal	Outlines 6 patient cases in which withdrawal was requested, either at the time or via an ADRT. Provide advice for HCPs to read current APM guidance, meet with the MDT, respect patients' wishes for timing where possible, have someone appropriate to administer medication (can be DN but specialist palliative care may be helpful to share decision making and assessment), have a separate room away from patient and family to prepare drugs (levels of medications for each case provided). Also, provide breakdown of time from decision to withdrawal, ventilator settings, time for overall process and time until death (this varied considerably from immediate to 33hrs).	Focus on withdrawal of NIV
Meyer et al. (2008). Elective termination of respiratory therapy in amyotrophic lateral sclerosis [39]	Germany	Retrospective cohort of *n* = 9 patients who had ventilation withdrawn	All took place in an inpatient setting. Describes the process of documenting the request for the withdrawal, the preparation and use of medications. For those with minimal or no spontaneous breathing deep sedation was preferred approach. Time until death was less for those receiving deep sedation. Lorazepam and diazepam used.	Focus on withdrawal.

Author and date	Country	Type of evidence/study method	Relevant findings	Limitations for the purpose of review
Moss et al. (1996) Patients With Amyotrophic Lateral Sclerosis Receiving Long-term Mechanical Ventilation*: Advance Care Planning and Outcomes [37]	USA	Structured interviews and QoL questionnaire with n = 50 patients	Patients had a mean Quality of Life score of 6.7/10. With those living at home more satisfied with life. A high proportion (79%) had an Advance Directive in place. 66% of those with an AD wrote in a preference of when to stop ventilation. Patients were most like to express these wishes and those for DNAR to their family (53% and 70%) rather than a physician (26% and 34%)	Age of paper. Predominance of TV, high rate of AD uptake may not be reflective of current patients
Oliver. (2004) Ventilation in motor neuron disease: Difficult decisions in difficult circumstances [56]	UK	Case illustration and discussion	Case of a woman with TV and progressively reduced communication. This was identified and she was asked if she wanted an AD in place. This was put in place to withdraw ventilation when she lost the ability to communicate. As this deteriorated she asked for withdrawal and this was undertaken. Some details of how and discussion of need for end of life conversations with MDT, patient and families.	Withdrawal of TV only.

**Author and date**	**Country**	**Type of evidence/study method**	**Relevant findings**	**Limitations for the purpose of review**
Phelps et al. (2017) Withdrawal of ventilation at the patient's request in MND: a retrospective exploration of the ethical and legal issues that have arisen for doctors in the UK [29]	UK	Interviews with *n* = 24 doctors involved in the withdrawal of ventilation	Focus on the ethical complexities of withdrawal. Doctors had a clear understanding of the legal position but found that despite this, withdrawal could be more challenging and could *feel* wrong. Wanted to discuss with others with more experience and to reach consensus within healthcare teams and families. Advance directives and clear instruction could be helpful but could also make it feel more uncomfortable. Legal and Trust bodies not often clear or helpful. A number of issues contribute to the complexity e.g. cause and effect, notion of planning to die, rarity, conscious patient, breathing as fundamental and relieving suffering.	Focus on withdrawal.
Phelps et al. (2022) Withdrawal of Assisted Ventilation at the Patient's request in MND: A retrospective exploration of the ethical and legal issues concerning relatives, nurses and allied health care professionals [51]	UK	Interviews with *n* = 17 bereaved family members and *n* = 26 nursing staff	Ethical and legal issues considered. Impact of these on the emotional stat of HCPs and family members. Need for guidelines. Confident HCPs who fully understand the legal position is essential. Impact of staff refusing to undertake withdrawal, on care, timing and place of death and family distress.	Focus on withdrawal

Author and date	Country	Type of evidence/study method	Relevant findings	Limitations for the purpose of review
Polkey et al. (1999) Ethical and clinical issues in the use of home non-invasive mechanical ventilation for the palliation of breathlessness in motor neurone disease [57]	UK	Review	Review of the use of NIV. One small section on the management of the terminal phase. Suggest patients may die of chest infection but doesn't give any further details. Touches on withdrawal and outlines key issues such as the need to be patient directed, the difference between withdrawal and euthanasia and the use of medications to support with complete removal or terminal weaning.	A small section on the management of the terminal phase. Age of paper may limited usefulness.
Thurn et al. (2019) Physicians' attitudes toward end-of-life decisions in amyotrophic lateral sclerosis [45]	Germany	Online survey using vignettes of European Physicians (*n* = 50) involved in MND care (Belgium, Germany, Ireland, Italy and UK)	Reports on findings from an online survey using vignettes. One vignettes used the example of an IV patient to assess attitudes to withdrawal. Only assesses if they would offer withdrawal and carry out withdrawal in those circumstances. Withdrawal was less likely to be offered and performed if the suffering was perceived to be physical rather than psycho-existential. This was likely to be affected by higher level of public religiosity and formal palliative care training. A more favourable approach was taken when any of the end of life measures were requested by the patient.	Very limited information on ventilation in relation to attitudes to withdrawal.

Author and date	Country	Type of evidence/study method	Relevant findings	Limitations for the purpose of review
Tripodoro et al. (2019) Withdrawing noninvasive ventilation at end-of-life care: is there a right time? [59]	Argentina	Review	Small sections touching on withdrawal, suggesting this is appropriate when it is no longer achieving the patient's goals. Identified gaps in knowledge around quality of dying and timely decision making.	MND only considered as part of neuromuscular diseases more widely. Primarily focused on acute use of NIV rather than ongoing use.
Turner et al. (2019) Tracheostomy in motor neurone disease [60]	UK	Review	Review of current information on TV including withdrawal. Sets out need for discussion and planning over time, potential to document wishes, effective management of symptoms during withdrawal, time until death needs to be communicated and planned for and that palliative care teams should be involved.	TV only

Author and date	Country	Type of evidence/study method	Relevant findings	Limitations for the purpose of review
Ushikubo. (2015) Comparison between home and hospital as the place of death for individuals with amyotrophic lateral sclerosis in the last stages of illness [48]	Japan	Interviews with home care nurses *n* = 14 in relation to *n* = 14 patient cases, recruited via a wider questionnaire survey to home care agencies.	10/14 had ventilation in place at time of death. However not of those using NIV were dependent. In 4/5 instances started as a life saving measure at end of life. More likely to die at home if have NIV and more likely to die in hospital if have TV. Also more like to have bulbar onset if die in hospital (probably because more likely to have TV). Time from diagnosis to death is longer for those in hospital (? Because more likely to have TV). Not clear what the 'education to prepare for death' was. Dying was not always recognised or discussed, a significant number had not had discussion about their wishes for ventilation. 2 were put on vent against their wishes and 2 without their wishes being known. Reports sudden and unpredicted deaths. 1 on TV died of cancer. 3/5 on TV death was not expected by family/home care worker.	Limited information on the deaths. Only 5 patients actually dependent on ventilation at the time of death.

Author and date	Country	Type of evidence/study method	Relevant findings	Limitations for the purpose of review
Ushikubo. (2018) Circumstances and Signs of Approaching Death in Patients With Amyotrophic Lateral Sclerosis Undergoing Noninvasive Ventilation in Home Care Settings [49]	Japan	Qualitative interview with *n* = 6 home visiting nurses followed by 3 group interviews each with 2 participants	Focus on nurses with experience of support MND patients with NIV who died in the last 3 years. Identified issues around levels of independence prior to death, lack of decision making, importance of keeping the limits of NIV in mind, repeated episodes of reviving. Some signs of approaching death were identified – repeated respiratory infection, weight loss, oral complications, skin breakdown, some respiratory distress. Some mentions of patients being prepared for death and the use of sedation. Also cites codes which identify 'accidental' deaths due to ventilation/use failures. The need for family caregivers in order to support ventilation use.	Not clear if the group participants were the same as those who took part in the original intervals. Poorly presented data, limited quotes and multiple 'codes'. Small sample.
Veronese et al. (2014) The last months of life of people with amyotrophic lateral sclerosis in mechanical invasive ventilation: A qualitative study. (Supporting information has been provided) [50]	Italy	Semi-structured interviews *n* = 19 with bereaved family caregivers	21% died suddenly. No reports of withdrawal. 47% at home. 11 stated they did not want TV, but 13 eventually did. Often this was due to no other choice, little time to decide or once the patient was unconscious. Findings show a number of predictors of death including loss of communication, clinical deterioration such as respiratory infections, need to medically manage symptoms such as pain, respiratory distress and agitation. These suggest there is preparation time and room for better management.	TV only. Patient perspectives reported by proxy.

Table S2. Data charting form - scope of findings

Very limited contribution to the review question

Some contribution to the review question

Useful focus on this issue contributing to the review question

					Withdrawal									
**Author**	**Place of death**	**Cause of death**	**Planning**	**Comment on death**	**Reason for WD**	**Timing of WD**	**Emotional Issues**	**Ethical/legal Issues**	**Practical Issues**	**Medication/ symptom**	**Pre-emptive sedation**	**Dosages**	**Who should be involved**	**Time until death**
AMP (2015) [25]														
Baxter et al. (2013) [13]														
Berger. (2012) [53]														
Burkhardt et al. (2017) [42]														
Cazzolli & Oppenheimer (1996) [47]														
Chapman et a. (2021) [52]														
Dreyer et al. (2012) [11]														
Eng. (2006) [61]														
Escarrabill et al. (2014) [40]														
Faull and Oliver. (2016) [58]														
Faull et al. (2014) [41]														
Faull and Wenzel. (2020) [46]														
Gannon. (2005) [54]														
Gleeson & Johnson. (2017) [17]														
Kettemann et al. (2017) [43]														
Kuhnlein et al. (2008) [38]														

Lebon & Fisher. (2011) [55]														
Markovic et al. (2018) [44]														
Messer et al. (2020) [18]														
Meyer et al.(2008). [39]														
Moss et al. (1996) [37]														
Oliver. (2004) [56]														
Phelps et al. (2017) [29]														
Phelps et al. (2022) [51]														
Polkey et al. (1999) [57]														
Thurn et al. (2019) [45]														
Tripodoro et al. (2019) [59]														
Turner et al. (2019) [60]														
Ushikubo. (2015) [48]														
Ushikubo. (2018) [49]														
Veronese et al. (2014) [50]														
